# Bridging innovation and risk: the missing role of validated risk-stratification in IgE-mediated food allergy management

**DOI:** 10.3389/falgy.2026.1848808

**Published:** 2026-07-02

**Authors:** Heather M. Opseth, Michelle Gruver

**Affiliations:** Doctor of Medical Science Department, Shenandoah University, Winchester, VA, United States

**Keywords:** biomarker, food allergy, immunoglobulin E (IgE), omalizumab, oral immunotherapy, risk-stratification, treatment

## Abstract

Over the last decade, the prevalence of IgE-mediated food allergies has increased, alongside therapeutic innovations such as oral immunotherapy (OIT), omalizumab, combination approaches, intranasal epinephrine, and emerging biomarker-guided strategies. This mini review synthesizes evidence published between 2019 and 2025 and evaluates how these advances intersect with persistent safety and clinical decision-making challenges. Although these therapies demonstrate promising efficacy, validated risk-stratification tools capable of guiding individual treatment selection are lacking. The proposed conceptual framework illustrates how patient characteristics, biomarkers, and treatment-specific risks may ultimately support individualized therapeutic decision-making.

## Introduction

Immunoglobulin E (IgE)–mediated food allergy (FA) affects up to 10% of the population and continues to impose substantial psychosocial, nutritional, and economic burdens (1). Traditional management has relied on strict allergen avoidance and rapid access to intramuscular epinephrine, strategies intended to reduce accidental exposure and treat reactions rather than induce immunologic tolerance (2).

Over the past decade, therapeutic innovation has expanded treatment options beyond avoidance alone. Oral immunotherapy (OIT), biologic agents such as omalizumab, and combination approaches are increasingly integrated into clinical care (3, 4). These advances mark a shift toward active, disease-modifying interventions, yet clinical decision-making has not evolved at the same pace. Despite increasing evidence supporting these therapies, standardized approaches capable of predicting which patients are most likely to benefit safely remain unavailable. Consequently, treatment selection often depends on subjective judgment, variable access to subspecialty care, and incomplete safety data.

IgE-mediated FA is associated with substantial morbidity and healthcare utilization (5). Most notably, patients who experience fatal allergic reactions often have a history of only mild prior symptoms, demonstrating that clinical history alone cannot predict risk (6). The absence of standardized, evidence-based algorithms leaves both patients and clinicians navigating complex decisions without clear risk guidance.

This mini-review synthesizes emerging evidence on innovative therapies for IgE-mediated FA and emphasizes the urgent need for validated risk-stratification strategies to ensure treatment progress translates into safer, more equitable, and effective care.

## Risk reduction strategies: avoidance, epinephrine advances, and biologics

Food allergen avoidance can effectively reduce reaction risks, but it is often challenging to implement. Difficulties involve the risk of accidental exposure, anxiety related to such exposure, modifications to socially acceptable dishes, avoidance challenges posed by common ingredients, mislabeling of packaged foods, and cross-contamination (2, 7).

While avoidance is not entirely risk-free, the danger mainly arises from accidental exposure. In such cases, the initial treatment of choice for anaphylaxis in IgE-mediated FA is epinephrine, most often given intramuscularly. Patient education is essential to ensure prompt identification of allergic reactions and immediate, effective epinephrine administration (7). Barriers to epinephrine use include dosing challenges, cost, obtaining a prescription, always carrying the medication, expiration, and misuse. When accessible and used properly, rapid intramuscular epinephrine use has been shown to decrease morbidity and mortality in patients with IgE-mediated FA anaphylaxis (8).

Given some of the challenges of intramuscular epinephrine, research has been conducted on alternative routes, including intranasal, sublingual, and inhaled (8). Intranasal epinephrine has shown promise in treating anaphylaxis. Studies have shown similar effectiveness to intramuscular injections and comparable, if not improved, tolerability and fewer contraindications (7, 9). Intranasal epinephrine may help reduce barriers such as ease of use, treatment delay, and fear of needles (9). There is variability in absorption with intranasal epinephrine requiring high doses to achieve similar plasma levels (7).

Omalizumab is another treatment option that could lower the risk for individuals with FAs. An injectable monoclonal antibody, omalizumab, binds to IgE, preventing the activation of mast cells and basophils, reducing the release of histamine and allergic reactions (10). Omalizumab has been shown to decrease the occurrence and severity of allergic reactions and is well-tolerated in those most studied with peanut and cow's milk allergy (10, 11). Although these studies demonstrate encouraging efficacy, most available investigations involve relatively small cohorts and heterogeneous populations, limiting generalizability. Variability in treatment protocols and follow-up duration further complicates reproducibility and hinders the development of standardized recommendations. Risk stratification tools could identify patients at an elevated risk of serious reactions from incidental exposure, inform treatment decisions, and provide guidance regarding long-term omalizumab therapy.

Treatment with omalizumab is not without potential risks. Omalizumab is generally well tolerated, with randomized controlled trials demonstrating adverse event rates comparable to placebo. Although infections have been reported, available evidence has not demonstrated a clinically significant increase in infection risk compared with controls (11). While there is no clear understanding of preferred populations to receive omalizumab treatment, some studies suggest that patients who have a positive oral food challenge and controlled comorbidities, such as asthma and eczema, are ideal candidates. There is still no consensus on the standard omalizumab treatment regimen, such as dosing, duration of use, and long-term safety (10). Food challenges are performed to assess a patient's response to biologic therapy, conferring additional risks from allergen exposure (5). Although omalizumab might assist with IgE-mediated FAs, it remains uncertain which patients are most likely to benefit. Qualitative research has demonstrated that both community and academic allergists report uncertainty regarding the integration of biologics into routine food allergy management. Common themes include variation in clinician experience, concerns regarding patient selection, and the absence of standardized guidance for determining which individuals are most likely to benefit from therapy (12). These findings further highlight the need for validated risk-stratification models and evidence-based decision-support tools to facilitate the safe implementation of biologic therapies. The development of evidence-based decision support tools is crucial to ensure that the benefits outweigh the risks in patients with IgE-mediated FA.

## Risk alteration: oral immunotherapy and biologic combinations

Oral immunotherapy (OIT) requires patients to follow an established protocol of daily, small but increasing amounts of allergen protein, such as peanut, cow's milk, or egg, to achieve desensitization and long-term unresponsiveness to allergens, thereby reducing the risk of future exposure. The end goal of OIT is for patients to consume the allergen without inciting an allergic reaction (13, 14). OIT studies report desensitization rates from 67% to 92%. While studies have shown benefits, the long-term duration remains largely unknown. OIT is only considered for pediatric patients, and research suggests that preschool-aged children have better results than older children undergoing OIT. Also, multiple OIT protocols exist without a standardized approach. While OIT appears effective in select populations, particularly for peanut, cow's milk, or egg allergies, the outcome on other or multiple food allergens remains unknown (13).

Safety is a critical consideration with OIT, and allergic responses are common, ranging from more common mild reactions to anaphylaxis. For this reason, OIT should be administered under the supervision of an allergist experienced in treating anaphylaxis (5). Adverse events are more likely to occur when the allergen protein dose is increased during OIT. Notably, studies have reported a higher prevalence of anaphylaxis in individuals undergoing OIT compared to those avoiding allergens (13). Illness and physical activity may increase the likelihood of an allergic reaction. While the severity of previous allergic reactions is not a contraindication for use, evidence recommends against OIT in patients with eosinophilic esophagitis or uncontrolled asthma (13). OIT could be a useful treatment for IgE-mediated FA, but more evidence is necessary to evaluate the risks and benefits for individuals. At present, no validated models reliably identify which patients are most likely to achieve sustained unresponsiveness, experience treatment failure, or discontinue therapy. The lack of predictive factors for responders and nonresponders further underscores the need for evidence-based risk-stratification tools. Identification of the patients who are at risk of anaphylaxis due to OIT therapy is essential to safely initiate therapy.

OIT combined with omalizumab has been proposed as a treatment approach in patients with IgE-mediated FA in an attempt to reduce the risk of allergic reaction. Buono et al. (3) performed a systematic review and meta-analysis, which revealed that OIT with omalizumab resulted in significantly higher desensitization rates, better-tolerated doses of allergen, and increased speed of reaching a maintenance dose. Omalizumab was found to decrease the rates of severe allergic reactions during OIT therapy (3).

Some information remains unknown, such as the long-term outcomes of OIT and omalizumab combined therapies. It is unclear what the optimal dose and duration of omalizumab are when used with OIT. Cost is another barrier to the addition of omalizumab with OIT (3).

Despite favorable desensitization rates, substantial heterogeneity exists among OIT protocols with respect to escalation schedules, maintenance dosing, and outcome definitions. This lack of standardization makes comparisons across studies difficult and limits translation into routine clinical practice. Furthermore, long-term sustained unresponsiveness remains incompletely understood, highlighting the need for prospective multicenter studies using harmonized protocols.

Identifying ways to predict a patient's risks related to OIT and omalizumab combined therapy is also necessary (3). Allergists have conflicting opinions on whether omalizumab should be used as monotherapy or combined with OIT (12). While this information supports the potential benefit of combination therapies, clinicians still lack data to help identify patients most likely to benefit while minimizing associated risk.

While therapeutic innovation continues to expand disease-modifying options for IgE-mediated food allergy, the ability to predict which patients are most likely to benefit safely from these therapies remains limited. Table 1 summarizes emerging therapeutic approaches, associated benefits, safety considerations, and the ongoing need for validated risk-stratification strategies in IgE-mediated food allergy management.

**Table 1:** Comparison of emerging IgE-mediated food allergy therapies and risk-stratification considerations.

Therapy	Population	Benefit	Key risks	Risk stratification need
OIT	Pediatric FA	Desensitization	Anaphylaxis	Identify ideal candidates
Omalizumab	Moderate/Severe FA	Reduced Reactions	Cost, infection	Determine high-risk patients
Combination Therapy	Select Patients	Improved Tolerability	Protocol variability	Predict responders

## Risk prediction: biomarkers and diagnostic tools

IgE serum levels can serve as an adjunct to diagnose IgE-mediated FA. IgE ≥ 0.35 kU_A_/L is the standardly accepted cut-off for a positive result, as supported clinically. Quantitative allergen-specific IgE concentrations are associated with an increased probability of positive oral food challenges and may provide clinically useful information when interpreted in the context of patient age and specific allergens. However, serum IgE concentrations do not reliably predict reaction severity (15). While serum IgE levels and trends are clinically monitored, in isolation they are insufficient for risk stratification. Nevertheless, when integrated with additional biomarkers and clinical characteristics, they may contribute to individualized treatment decision-making.

Age-specific analyses have demonstrated that increasing allergen-specific IgE concentrations are associated with a higher probability of positive food challenges, with threshold values differing among younger and older children. These findings suggest that quantitative IgE measurements may contribute to treatment decision-making when interpreted alongside other patient characteristics and biomarkers (16).

While not currently used routinely in clinical practice, several emerging biomarkers are being investigated to improve risk assessment in IgE-mediated food allergy. The basophil activation test (BAT) is one such assay that evaluates basophil responsiveness following exposure to specific food allergens. Preliminary studies suggest that BAT may help predict reaction severity during oral food challenges and has demonstrated high sensitivity and specificity in peanut allergy. However, much of the available evidence originates from single-center studies with relatively small sample sizes, and it remains uncertain whether these findings can be generalized to other food allergens (15).

Similarly, IgG4/sIgE ratios have been investigated as markers of developing tolerance, with higher ratios associated with greater allergen tolerance in some studies (17). Despite their promise, significant barriers to clinical implementation remain. Variability in laboratory methodologies, lack of standardized thresholds, limited availability, and the absence of large multicenter validation studies hinder widespread adoption. Consequently, these biomarkers currently serve primarily as investigational tools rather than clinically actionable measures. Future research should prioritize standardized assays and external validation to facilitate incorporation into evidence-based risk-stratification models and individualized treatment algorithms.

## Decision model

Given the increase in novel IgE-mediated FA therapies, patients and/or families now have therapy options rather than exclusive avoidance. This shift now requires increased shared decision-making between patients and providers. Anagnostou et al. (18) developed a clinical decision-making tool to aid caregivers of pediatric patients in the selection of the appropriate IgE-mediated FA treatments, taking into consideration food allergy burden, available treatment options (OIT and avoidance only), considerations, and concerns. The study found sufficient acceptance of the tool by caregivers. The tool did not consider biologic treatments as monotherapy or OIT adjunct. Further, the tool does not assess the individual risk of seeking various treatments (18). Patients and/or family members need risk stratification as part of the clinical decision-making process, which currently does not exist. However, this existing clinical decision-making tool could lay the foundation for a future instrument that includes risk assessment to predict the likelihood of serious patient outcomes.

Although caregiver acceptance of decision aids appears favorable, practical implementation challenges remain. Existing tools have not undergone large-scale external validation, and incorporation into clinical workflows may require integration with electronic health records, standardized outcome reporting, and clinician training. These barriers illustrate the complexity of translating conceptual frameworks into scalable clinical decision-support systems.

## Methods

A structured literature search was performed in PubMed, Cochrane Library, and WorldCat for articles published between January 2019 and January 2025 to capture developments from the past decade of rapid therapeutic expansion.

Search terms included: “food allergy,” “IgE-mediated,” “oral immunotherapy,” “omalizumab,” “biologic therapy,” “biomarkers,” “risk,” “risk-stratification,” and combinations using Boolean operators such as “oral immunotherapy AND omalizumab” and “food allergy AND biomarkers.” Table 2 summarizes search terms and Boolean combination examples. Reference lists of key articles and recent systematic reviews were also screened to identify additional relevant studies.

**Table 2:** Search strategy in Cochrane Library, WorldCat, and PubMed.

Search terms	“food allergy,” “IgE-mediated,” “oral immunotherapy,” “omalizumab,” “biologic therapy,” “biomarkers,” “risk,” “risk-stratification”
Boolean combination examples	“omalizumab AND oral immunotherapy,” “food allergy AND biomarkers”

Because this review synthesizes previously published literature without involving human subjects, Institutional Review Board (IRB) approval was not required. Findings were integrated narratively to highlight emerging therapeutic themes, identify limitations in current clinical decision-making, and underscore the urgent need for validated risk-stratification tools in IgE-mediated food allergy management.

## Future directions and discussion

The rising prevalence of IgE-mediated food allergy underscores the growing urgency to close the critical gap surrounding treatment selection and patient safety. Although immunotherapies, biologics, and emerging biomarkers continue to expand therapeutic possibilities, these interventions carry heterogeneous risks that remain difficult to predict (3). Without validated risk-stratification tools, clinicians and families must make high-stakes decisions based largely on clinical judgment, incomplete evidence, and variable experience rather than standardized, data-driven guidance. Research has identified variables that must be considered in therapy decision-making, including clinical history, evidence of effectiveness, patient preferences, availability of therapy, cost, acceptability, feasibility, and equity (19).

**Figure 1:**
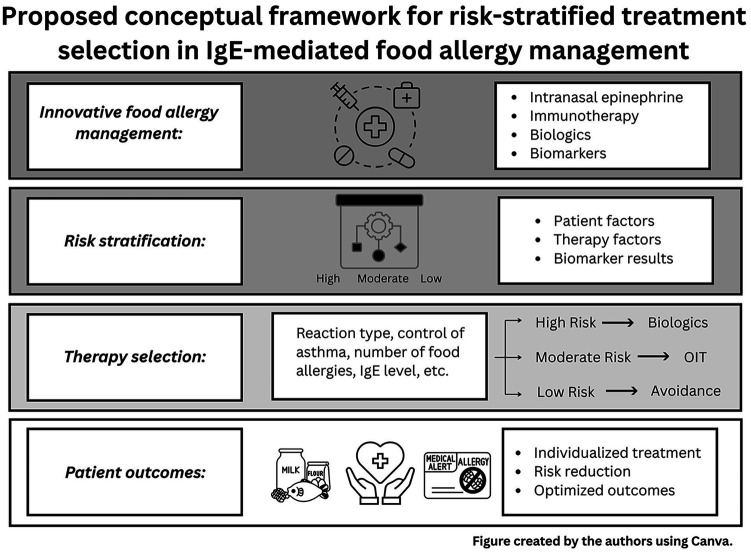
Proposed conceptual framework for risk-stratified treatment selection in IgE-mediated food allergy management.

Experts note that choosing FA therapy is not a one-size-fits-all approach (4). A validated decision-support tool should examine patient age, comorbidities, including control status, reaction histories, number of food allergies, therapy risks, specific food allergen(s), IgE biomarker findings, and BAT thresholds. This data could help classify patients into a three-tier risk model, such as low, moderate, and high risk. Based on this categorization, a guiding hypothesis could be that high-risk patients receive biologics, moderate-risk patients undergo OIT, and low-risk patients engage in avoidance as depicted in Figure 1. This hypothesis could be tested through close monitoring of safety outcomes.

Future research should prioritize multicenter collaborations capable of generating large, diverse datasets to identify predictors of treatment tolerance, adverse reactions, and long-term outcomes. An example of an algorithm for FA treatment includes required dietary modifications, psychological support, and emergency treatment plans. While immunomodulatory treatments have been considered, it does not clearly specify which treatments are preferred vs. higher risk in specific patient populations (19). While research has focused on the efficacy of traditional avoidance, immunotherapy, biologics, biomarkers, and decision-making tools, future investigations must specifically examine them through the lens of risk stratification. Another important challenge involves standardization across institutions. Differences in outcome measures, adverse event reporting, and treatment protocols hinder comparisons among studies and slow the development of reliable predictive models. Establishing multicenter collaborative networks and consensus reporting standards would facilitate external validation and improve the reproducibility of future research. Emerging technologies, including multi-omic profiling and machine learning-assisted prediction models, may further improve future risk prediction and precision treatment selection. An important limitation of the current literature is the predominance of systematic reviews, consensus statements, and expert opinion documents, reflecting the relatively early stage of therapeutic development. Additional prospective studies with standardized outcomes are needed to generate the primary evidence required for robust risk-stratification models.

Additionally, the field would benefit from standardized reporting of adverse events, harmonization of outcome measures across clinical trials, and intentional study of populations historically underrepresented in food allergy research. Institutions could adopt standardized reporting techniques to enhance data collection and usability. As treatment approaches become increasingly complex, incorporating patient values, cost considerations, access constraints, and health equity principles into future algorithms will be essential. Implementation science methodologies may play an important role in bridging the gap between discovery and practice.

Future risk-stratification tools must not only demonstrate predictive accuracy but also prove feasible, acceptable, and sustainable within real-world allergy clinics. Integration with electronic medical records and shared decision-making frameworks may enhance clinical adoption and maximize patient-centered care. Finally, implementation science strategies should be used to ensure that any validated tools can be effectively integrated into clinical workflows and shared decision-making. The development and validation of clinically applicable risk-stratification models represent a critical next step in ensuring that rapid therapeutic innovation translates into safer, more equitable, and increasingly personalized care for individuals with IgE-mediated food allergy.
